# Authors’ Reply: The Power of Collaboration in Facilitating Mobile Technology Adoption in Health Care

**DOI:** 10.2196/62891

**Published:** 2024-07-08

**Authors:** Huong Ly Tong, Severin Rakic, Hazzaa M Al-Hazzaa, Saleh A Alqahtani

**Affiliations:** 1 Cultural and Indigenous Research Centre Australia Redfern Australia; 2 The World Bank Group Washington, DC United States; 3 Lifestyle and Health Research Center Health Sciences Research Center Princess Nourah Bint Abdulrahman University Riyadh Saudi Arabia; 4 School of Sport Sciences University of Jordan Amman Jordan; 5 Organ Transplant Center of Excellence King Faisal Specialist Hospital & Research Center Riyadh Saudi Arabia; 6 Division of Gastroenterology and Hepatology Weill Cornell Medicine New York, NY United States

**Keywords:** mobile apps, fitness trackers, SMS, SMS text messaging, physical activity, exercise, sedentary behavior, Middle East, Africa, Northern, movement, physical inactivity, smartphone, mobile phone, mobile health, mHealth, digital health, behavior change, intervention

We are responding on behalf of our author group to a letter to the editor [1] regarding our recent article titled “The Use of Mobile Technologies to Promote Physical Activity and Reduce Sedentary Behaviors in the Middle East and North Africa Region: Systematic Review and Meta-Analysis” [2], published in the *Journal of Medical Internet Research*. We thank the letter’s authors for their interest in our article and the journal editor for providing us the opportunity to respond. Our response is limited to the 4 aspects of critique in the letter.

First, regarding the database search, we agree with the letter’s authors that the more databases searched, the more comprehensive the results would be. In our review, we conducted a search across 5 major databases, including MEDLINE, Embase, CINAHL, Scopus, and Global Index Medicus. This is in line with the standard practices outlined by the Cochrane Handbook for Systematic Reviews of Interventions, which recommends using at least 3 databases [3]. Given the focus of the review on the Middle East and North Africa, we used Global Index Medicus for our search to enhance comprehensiveness. Moreover, a gray literature search was also conducted, in Google Scholar, to ensure that we identified the relevant studies. Paradoxically, the letter’s authors referred to a systematic review [4] that used fewer databases in their search than we did to argue the database search point.

Second, we agree that frequency and duration of the intervention are important aspects. Indeed, we analyzed this information and discussed it in Tables 1 and 2 of our article, as well as in the main text: “The average duration of experimental studies was 20 (SD 14.4; range 6-52) weeks.” and “Half (11/22, 50%) of the interventions used SMS text messaging to deliver educational content…; the frequency of delivery varied from 2 messages per day to 1 message per week” [1].

Third, we agree with the letter’s authors that potential confounding factors can influence the findings of the reported studies. However, we found that important confounders were not reported in the many studies reviewed. While we planned to explore the role of confounders via planned meta-regression [5], it would have been methodologically flawed to do so, given that the meta-analysis would have been based on 7 studies only. At least 10 studies should be available for each characteristic modeled [3]. The influence of some reported confounders was discussed in the article, including socioeconomic status, context fit, and cultural appropriateness.

Finally, we agree with the authors that social workers can facilitate mobile technologies deployment. However, exploration of the social workers’ role in this process was not an objective of the review nor a primary focus of the articles included in our review.

We would welcome future publications that discuss specifically the role of social workers in the deployment of mobile technologies.
